# Specific microRNAs Regulate Heat Stress Responses in *Caenorhabditis elegans*

**DOI:** 10.1038/srep08866

**Published:** 2015-03-09

**Authors:** Camilla Nehammer, Agnieszka Podolska, Sebastian D. Mackowiak, Konstantinos Kagias, Roger Pocock

**Affiliations:** 1 Biotech Research and Innovation Centre, University of Copenhagen, Ole Maaløes Vej 5, Copenhagen, Denmark; 2 Max-Delbrück-Center for Molecular Medicine, Robert-Rössle-Straße 10, 13125, Berlin; 3 Department of Anatomy and Developmental Biology, Faculty of Biomedical and Psychological Sciences, Monash University, Clayton, Victoria, Australia

## Abstract

The ability of animals to sense and respond to elevated temperature is essential for survival. Transcriptional control of the heat stress response has been much studied, whereas its posttranscriptional regulation by microRNAs (miRNAs) is not well understood. Here we analyzed the miRNA response to heat stress in *Caenorhabditis elegans* and show that a discrete subset of miRNAs is thermoregulated. Using in-depth phenotypic analyses of miRNA deletion mutant strains we reveal multiple developmental and post-developmental survival and behavioral functions for specific miRNAs during heat stress. We have identified additional functions for already known players (*mir-71* and *mir-239*) as well as identifying *mir-80* and the *mir-229 mir-64-66* cluster as important regulators of the heat stress response in *C. elegans*. These findings uncover an additional layer of complexity to the regulation of stress signaling that enables animals to robustly respond to the changing environment.

Animals are continually exposed to changing environmental conditions. For the nematode *C. elegans*, its ephemeral natural habitat fluctuates rapidly where worms at different stages of development need to sense and respond to changes in pH, ultraviolet light, respiratory gases, temperature and many other environmental factors^1^,^2^. In order to combat these environmental stressors, *C. elegans* has evolved multiple stress-protective responses that include aversive behavioral strategies, instigation of alternative developmental programs (dauer entry), in addition to metabolic adjustments^3^,^4^,^5^,^6^,^7^,^8^,^9^. Some of these survival tactics are mediated by conserved transcriptional regulators; such as, the hypoxia-inducible factor HIF-1 to mount responses to hypoxic insults^7^, DAF-16/FOXO to regulate metabolism and dauer formation^10^ and the heat-shock transcription factor HSF-1 to combat heat stress^11^. In addition, these transcription factors perform overlapping functions during stress responses and lifespan control^12^,^13^,^14^. The targets and downstream pathways controlled by these conserved transcription factors during stress have been extensively studied and have provided many important insights to the area of stress biology. However, much less is known of the roles of posttranscriptional regulators in the control of environmental stress responses.

microRNAs (miRNAs) are a class of short noncoding RNAs that operate as key posttranscriptional regulators of eukaryotic gene expression. miRNAs predominantly regulate gene expression through imperfect base-pairing with sites harbored in the 3′UTR regions of their mRNA targets^15^,^16^. Computational prediction programs identify hundreds of putative targets for each miRNA, the regulation of which may be dependent on the temporal, spatial and/or environmental context of a particular cell or tissue. The first miRNAs to be discovered, *lin-4* and *let-7*, were shown to control the timing of specific events during *C. elegans* development^17^,^18^,^19^,^20^. Since then, *C. elegans* miRNAs have been shown to control a wide array of biological processes including embryonic development, cell-fate specification, axon guidance, physiology and longevity^21^,^22^,^23^,^24^,^25^,^26^,^27^,^28^,^29^. However, most *C. elegans* miRNAs have no assigned function^30^ and there is a paucity of information regarding the overall role of miRNAs in the response to environmental stressors.

In this study, we focused on the roles of miRNAs in the regulation of the heat stress response. We hypothesized that miRNAs regulated during heat stress may be important for the ability of animals to perform normal physiological functions (locomotion, reproduction and survival) in elevated temperatures. To this end, we performed high-throughput sequencing to identify thermoregulated miRNAs. Then we obtained deletion alleles for these miRNAs and identified four strains containing deletions in individual (*mir-71*, *mir-80* and *mir-239*) or multiple miRNAs (*mir-229 mir-64-66*) that are required for appropriate responses to heat stress. Using detailed phenotypic analyses where we exposed animals at different stages of development to varying levels of environmental temperature, we identified an array of embryonic and postembryonic developmental decisions, including lifespan, which are controlled by a subset of thermoregulated miRNAs (TRMs). We performed molecular analysis to ask whether known heat stress responsive pathways (HSRs) are affected in thermoregulated miRNA mutant backgrounds and found that these pathways are not significantly perturbed. This suggests that thermoregulated miRNAs act in other pathways or downstream of the HSRs we analyzed to enable appropriate heat stress responses. We propose that the posttranscriptional control of a combination of regulatory pathways by thermoregulated miRNAs afford *C. elegans* robustness when confronted with fluctuations in environmental temperature.

## Results

### Multiple miRNAs are Thermoregulated in *C. elegans*

**Figure 1:**
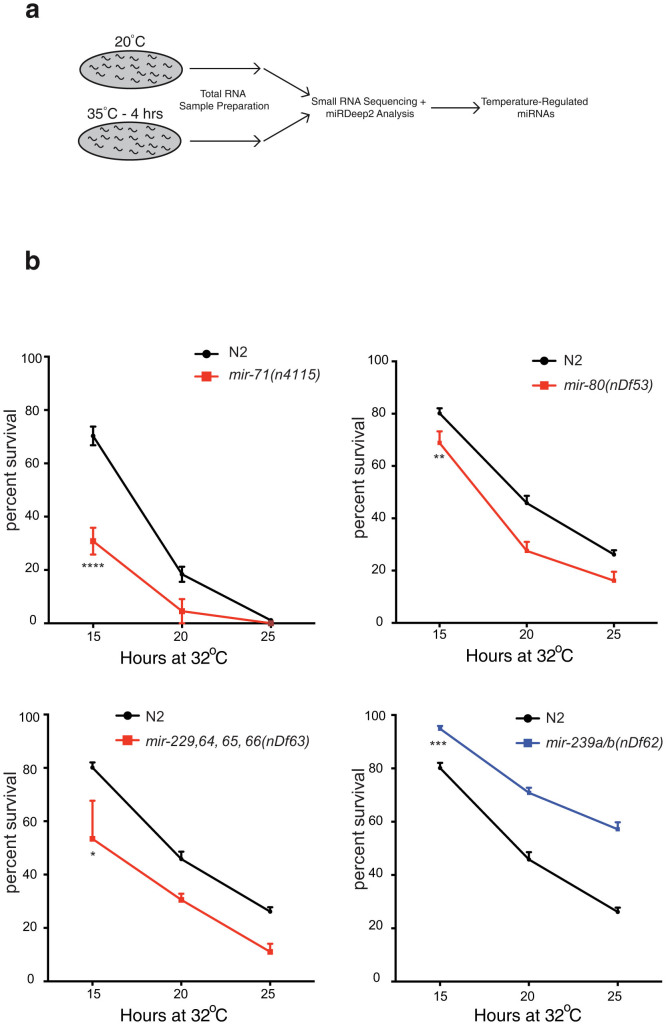
Specific miRNAs are Important for the Heat Stress Response. (a) Synchronized L4 wild type animals were exposed to 35°C for 4 hrs. Total RNA was isolated from this sample in addition to aged-matched control animals cultivated at 20°C. Both RNA samples were sequenced on the Illumina platform and analyzed with miRDeep2. (b) Deletion mutants of differentially expressed miRNAs affect survival at 32°C. Each mutant strain was incubated at 32°C for 15, 20 and 25 hrs and recovered for 24 hrs at 20°C. We used adult animals for these assays, and not L4 larvae as were used in the RNA sequencing, as heat stress assays using L4 animals are inconsistent in our hands. Heat stress assays were performed in triplicate. Data are presented as means ± SEM and statistical significance was assessed by 2way ANOVA followed by Tukey's multiple comparison test. n > 110, *p < 0.05, **p < 0.01, ***p < 0.001, ****p < 0.0001.

To discover miRNAs with potential functions in heat stress responses, we first sought to identify miRNAs that are regulated at high temperature. We hypothesized that such thermoregulated miRNAs may be important for the control of survival mechanisms and other physiological functions during heat stress. To identify miRNAs regulated by heat stress, we exposed populations of synchronized larval stage 4 (L4) wild type N2 animals to 20°C and 35°C for 4 hrs and isolated total RNA (Figure 1A). We performed high-throughput small RNA sequencing on the Illumina platform followed by miRDeep2 analysis to identify miRNAs regulated by an elevation in temperature^31^,^32^. We obtained a total of 12,912,387 and 9,503,977 raw reads corresponding to 5,017,174 and 3,102,595 mature miRNA reads from the 20°C and 35°C samples, respectively. Using a > 1.9-fold in expression change and a minimum of 150 normalized read count cut-off we identified 19 miRNAs regulated by temperature (Table 1 and Table S1). We noticed that the majority of miRNAs (13 out of 19) with >1.9-fold change in expression were downregulated in heat stress. This could indicate that components of the miRNA biogenesis machinery may be downregulated in these conditions. Using quantitative real-time PCR (qRT-PCR) we found that transcript levels of the major miRNA biogenesis genes *drsh-1*/*Drosha* and *dcr-1/Dicer* are stable after 4 hrs of heat stress and the levels of *alg-1/Argonaute* drop by about 40% after 2 hrs (Figure S1). This reduction of *alg-1* levels may be required to reduce miRNA levels to enable high expression of target genes. However, such broad regulation would be an inherently unspecific means of controlling miRNA expression, therefore we postulate that other regulatory mechanisms exist to control the levels of specific miRNAs during heat stress.

### Specific miRNAs Regulate Organismal Survival at High Temperature

Out of the 19 miRNAs regulated by heat stress in *C. elegans* (Table 1 and Table S1), we obtained strains that harbored miRNA deletions for 14 of them^30^. To test whether these miRNAs have essential roles in high temperatures, we performed longitudinal heat stress assays at 32°C. We exposed synchronized young adults of each miRNA mutant strain to 32°C for 15, 20 and 25 hrs and compared the ability of miRNA mutant and wild type N2 animals to survive heat stress. We found that 4 of the 14 miRNA mutant strains we tested exhibited a significant change of sensitivity or resistance to heat stress when compared to wild type and are rapidly regulated in heat stress (Figures 1 and S1). We found that loss of *mir-71*, *mir-80* and the *mir-229 mir-64-66* cluster causes heat sensitivity and loss of *mir-239* causes heat resistance (Figure 1B). We confirmed thermoregulation of all these miRNAs at the lower 32°C with the exception of *mir-239,* which therefore may not need to be regulated to perform its role at this temperature (Figure S1). Roles for *mir-71* and *mir-239* in regulating the heat stress response have previously been reported^23^,^26^. Heat stress phenotypes for *mir-80* and *mir-229 mir-64-66* mutant animals were rescued by transgenic array harboring a wild type copy of the respective miRNA locus, however, we were unable to rescue the *mir-239a/b* heat resistance phenotype (Figure S2 and data not shown). Finally, heat sensitivity caused by loss of *mir-71* was previously rescued in another study^23^*.*

### Lifespan Control by Thermoregulated miRNAs

**Figure 2:**
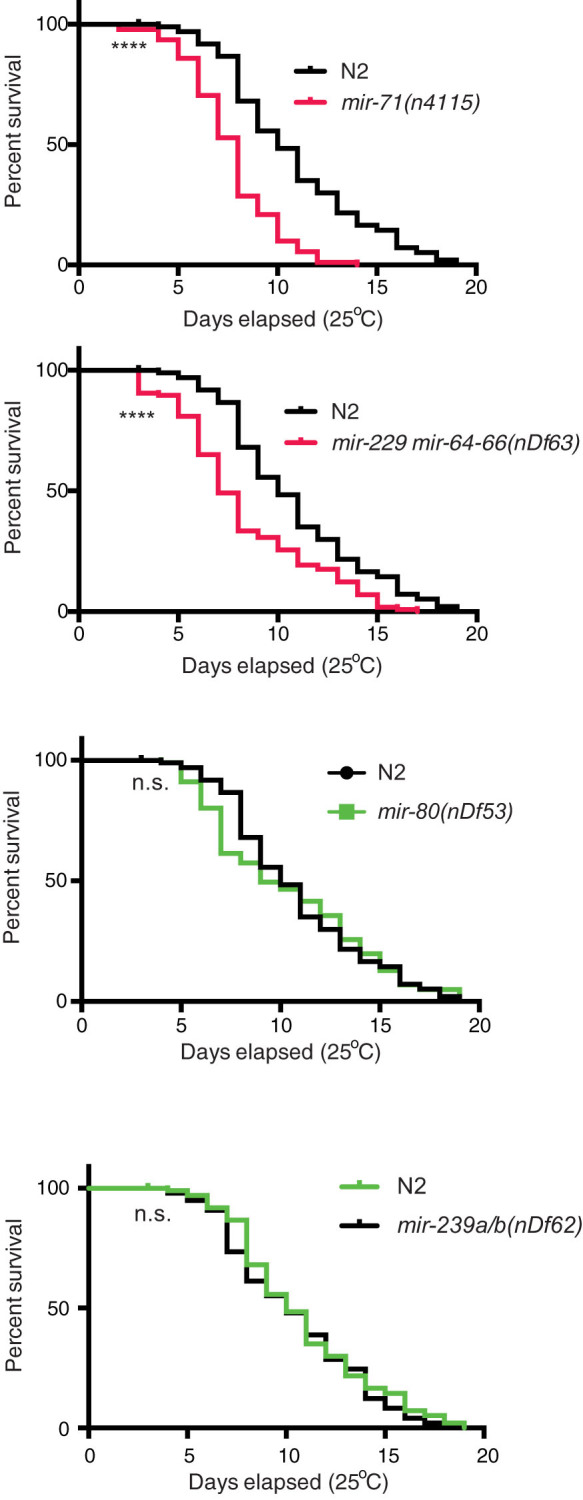
Specific miRNAs Function to Control Lifespan at 25°C. *C. elegans* mutants that contain deletions of *mir-71(n4115)* and *mir-229 64-66(nDf63)* exhibit significantly reduced lifespan compared to wild type N2 animals (black), whereas *mir-80(nDf53)* and *mir-239(nDf62)* deletions do not. lifespan analysis was repeated twice (n > 90–118) with similar effects (Table S2). Mean lifespan values and statistical analyses of lifespan assays are shown in Table S2. ****p < 0.0001, value refers to the experimental strain and corresponding wild type control animals. n.s. - not significant.

The link between miRNA control of stress responses and their regulation of lifespan is well established^11^,^26^. Certain *C. elegans* mutants that are sensitive to a variety of stresses, for example *mir-71*, are short-lived whereas, stress resistance endows a longer life, as is the case for *mir-239*^23^,^26^. We hypothesized that the four TRMs in which we identified heat stress phenotypes, may exhibit altered lifespan. Therefore, we performed longevity assays and found that indeed *mir-71* and *mir-229 mir-64-66* mutant strains are short lived at 25°C (Figure 2 and Table S2). Previous work has shown that loss of *mir-80* or *mir-239* extends lifespan at 20°C^26^,^27^, however this positive effect on lifespan was not observed in our experiments at the more stressful 25°C temperature (Figure 2 and Table S2). Taken together, these observations show that *mir-71* and *mir-229 mir-64-66* mutant animals are short-lived at 25°C in addition to being heat stress sensitive.

### *C. elegans* Reduces Motility at Elevated Temperature

**Figure 3:**
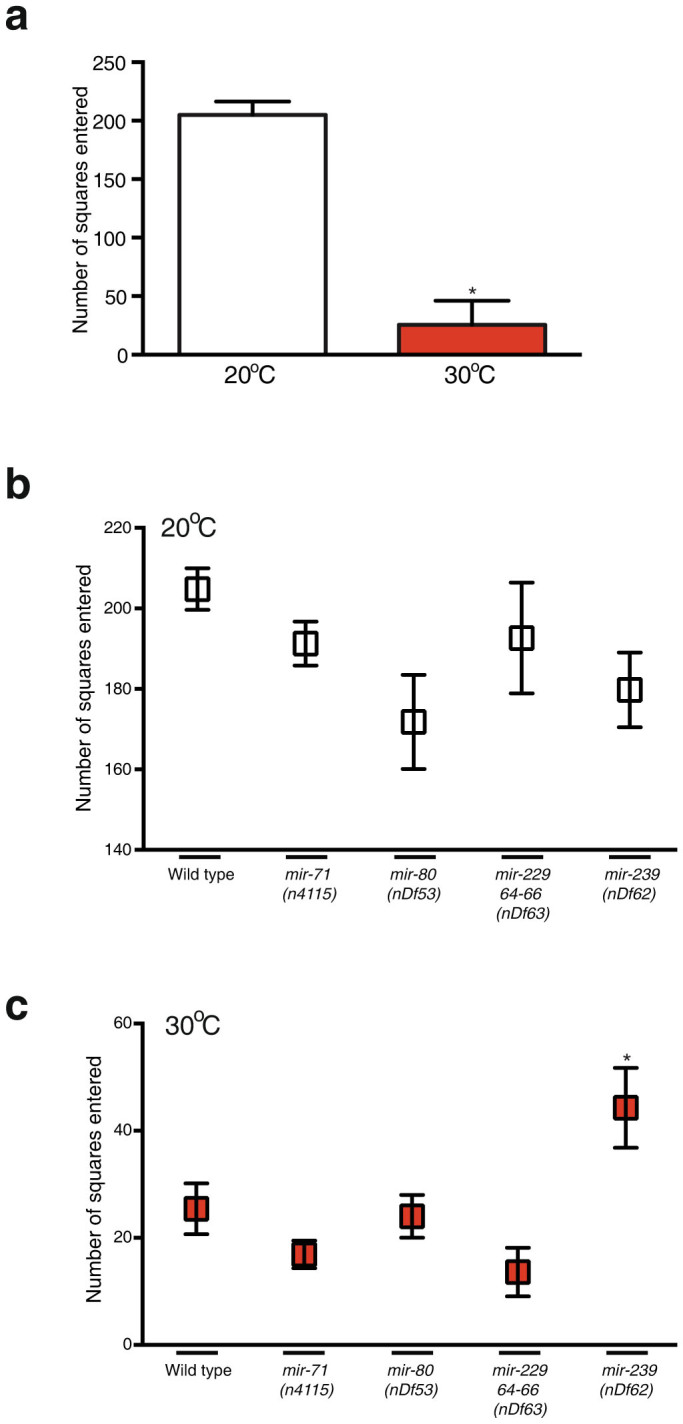
*C. elegans* Reduces Motility in Elevated Temperature. (a–c) Synchronized L4 animals were exposed to 20°C or 30°C for 6 hrs, followed by 16 hrs on plates fully covered with bacteria to assay motility. Data is presented as number of squares the worm crossed within 16 hrs out of a total of 240 squares. Data are presented as means ± SEM and statistical significance was assessed by ANOVA followed by Dunnett's multiple comparison test n > 13, *p < 0.05. The wild-type worms presented in (a) are the same as for (b) for illustration.

It has been shown that reduction of energy metabolism extends lifespan and stress resistance in *C. elegans*^33^,^34^. One way of reducing energy demand would be to reduce motility. We tested this hypothesis by placing single worms on fully coated bacterial plates at either 20°C or 30°C for 16 hrs and measured their motility (Figure 3). We found that wild type animals reduce their motility by almost 10-fold at 30°C (Figure 3A). We next tested whether TRM mutant strains exhibit altered motility at either 20°C or 30°C when compared to wild type. We found that all strains behaved like wild type except for the *mir-239* mutant, which showed and increase in motility compared to wild type at 30°C (Figure 3). The reason for this increase in motility in the *mir-239* mutant is unclear and would require further study.

### miRNAs Control Egg-Laying Rate in Response to Change in Temperature

**Figure 4:**
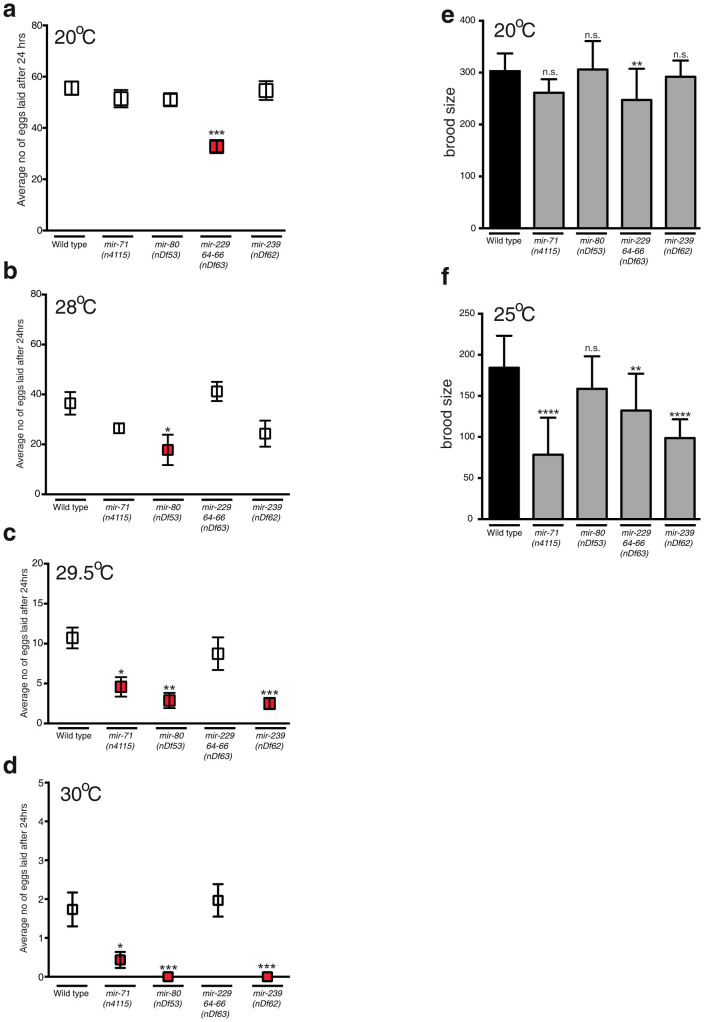
Egg-Laying Rate and Brood Size are Controlled by miRNAs During Heat Stress. (a–d) Egg-laying proficiency of young adults within a 24-hr period was assayed at the following temperatures: 20°C (a), 28°C (b), 29.5°C (c) and 30°C (d). Data are presented as means ± SEM and statistical significance was assessed by one-way ANOVA analysis followed by Dunnett's multiple comparisons test. n > 10 for 20°C and 28°C, for all other assays n > 28, *p < 0.05, **p < 0.01, ***p < 0.001, n.s - not significant. Experiments were performed in triplicates on three independent days. (e–f) Brood sizes were scored at 20°C and 25°C. Data are presented as means ± SEM. Statistical significance was assessed by one-way ANOVA analysis followed by Dunnett's multiple comparisons test. n > 12, **p < 0.01, ****p < 0.0001 n.s. - not significant. Experiments were performed in triplicates on three independent days.

A previous study found that egg-laying proficiency is sensitive to changes in temperature^35^. At 28°C, wild type animals robustly lay eggs, whereas at 30°C egg-laying is almost completely abrogated^35^. We investigated whether TRMs regulate this behavior as we have shown in the present study that these miRNAs are regulated rapidly at the L4 stage of development, which would be an appropriate stage to make such an important behavioral decision. We first tested the ability of TRM mutant adults to lay eggs in a 24-hr period at 20°C and found that loss of *mir-229 mir-64-66* caused a slight drop in egg-laying rate (Figure 4A), suggesting a role for these miRNAs in the regulation of egg-laying. At 28°C, all strains exhibited a decrease in the number of eggs laid with *mir-80* animals laying significantly fewer eggs than wild type (Figure 4B). As previously published^35^, at 30°C egg-laying is severely affected where very few eggs are laid by wild type animals in 24 hrs (Figure 4D). We did however observe an additional drop in egg-laying in *mir-71* mutant animals and a complete abrogation of egg-laying in *mir-80* and *mir-239* mutant animals at 30°C. Remarkably, when we performed the same experiment but decreased the incubation temperature by 0.5°C to 29.5°C, wild type animals lay 10-fold more eggs than at 30°C (Figure 4C). In these conditions, *mir-71*, *mir-80* and *mir-239* mutant animals still laid significantly fewer eggs than wild type. These experiments show that egg-laying in *C. elegans* is acutely sensitive to small changes in environmental temperature and that *mir-71*, *mir-80* and *mir-239* may regulate this response.

### Specific Thermoregulated miRNAs Control Brood Size

We have shown that specific miRNAs control temperature-regulated egg production. This could possibly be a means of preventing the generation of progeny in unfavorable conditions. We next sought to better understand whether these miRNAs play more general roles in regulating responses to heat stress by analyzing progeny production in less harsh environments. First, we found that the brood sizes of TRM mutants were not significantly different to wild type animals at 20°C, with the exception of *mir-229 mir-64-66* which has a small decrease in brood size (Figure 4E). We then exposed TRM mutant L4 animals and their resultant progeny to 25°C throughout development. We observed that loss of *mir-71*, *mir-229 mir-64-66* and *mir-239* caused significant decrease in brood size compared to wild type animals at 25°C, yet caused no embryonic lethality (Figure 4F and data not shown), suggesting that these miRNAs are required to regulate gamete production in high environmental temperatures.

### Canonical Heat Stress Response Pathways Are Largely Unaffected in Thermoregulated miRNA Mutants

We postulated that the phenotypes observed during heat stress in TRM mutant animals could be caused by disruption of conserved genetic pathways required for the heat stress response. To test this hypothesis, we quantified the induction of heat shock protein (HSP) and *hsf-1* expression (Figure S3). We employed qRT-PCR to assay the constitutive and heat-shock induced expression of HSP and *hsf-1* mRNA levels (Figure S3). These assays were normalized using two reference genes (*gpd-1* and *gpd-4*) that are invariant at 32°C compared to the control temperature of 20°C. As shown previously^36^, we found that in wild type animals, expression of *hsp-16.1*, *hsp-16.2* and *hsp-70* is rapidly induced after 1 hr of heat stress at 32°C, whereas the expression of *hsf-1* is upregulated after 1 hr, after which levels drop back to baseline (Figure S3A). To test whether TRM regulated pathways control the expression of these factors at the transcriptional level we assayed their expression after 1 hr of heat stress at 32°C. We found that the transcript levels of the heat shock proteins and *hsf-1* are induced after heat stress in the TRM mutant backgrounds at a similar level to wild type animals (Figure S3B–E). Therefore, transcription of major heat stress response genes is induced at normal levels in TRM mutant animals.

## Discussion

In this study we have revealed an additional layer of genetic regulation that controls organismal heat stress responses. Using high-throughput sequencing analysis of miRNA expression during heat stress as well as phenotypic analysis of genetic deletion strains, we identified miRNAs that control both survival and behavior during heat stress. These miRNAs act during both embryonic and adult life to modify *C. elegans* responses to environmental temperature perturbations. We have identified additional functions for already known players of the heat stress response (*mir-71* and *mir-239*) as well as identifying two miRNAs (*mir-80* and the *mir-229 mir-64-66* cluster) that impact on the heat stress response in *C. elegans*.

In post-developmental adult life, *C. elegans* requires a variety of metabolic and behavioral strategies to cope with increased environmental temperature. The primary goal of a *C. elegans* hermaphrodite is to produce the maximal number of offspring in conditions that are conducive to embryonic development and survival. We found that *mir-71*, *mir-80* and *mir-239* are required for generation of embryos in elevated temperatures. Genetic deletion of these miRNAs causes a reduction of eggs laid at high temperatures, suggesting two non-mutually exclusive scenarios. First, loss of a specific miRNA may render the gonadal system sensitive to elevated temperature and thereby prevent egg-laying. Second, embryos lacking specific miRNAs do not survive well at elevated temperatures; therefore, miRNA mutant hermaphrodites may detect a metabolic change in the gonad, due to loss of a miRNA, and then prevent, or slow down, oocyte production in unfavorable conditions.

A different behavioral strategy that animals may use to cope with detrimental thermal conditions would be to reduce energy expenditure by decreasing their motility. Indeed, we found that wild type animals reduce their motility by 10-fold when switched from 20°C to 30°C. None of the TRM mutant strains affected this behavioral switch except for the *mir-239* mutant, which exhibits almost twice as much motility compared to wild type animals at 30°C but not at 20°C. Therefore, *mir-239* mutants potentially cope better with higher temperatures and their motility is not affected under such stressful conditions or that they lack the motor response mechanism required to drive this behavior.

Previous work has identified miRNAs that are required for lifespan regulation at 20°C^23^,^25^,^26^,^27^,^29^. To ask whether TRMs play roles in regulating longevity in more stressful conditions we performed lifespan experiments at 25°C (following developmental incubation at 20°C). In these experiments, we confirmed that *mir-71* and *mir-229 mir-64-66* are required for normal lifespan at 25°C as they are at 20°C. Other miRNAs shown to regulate lifespan at 20°C, such as *mir-80* and *mir-239*, exhibited no altered lifespan phenotype at 25°C. Our sequencing data indicate that *mir-80* and *mir-239* are regulated at elevated temperatures. Such lack of regulation in the mutant strains at 25°C may therefore negate lifespan extension at this temperature.

We found that the degree and direction of expression change of TRMs is not necessarily a predictor of their roles in heat stress. Many TRMs for which we uncovered heat-related phenotypes were downregulated in heat stress. Such downregulation does not seem to directly associate with the phenotypic consequences observed. There are a number of reasons why this would be the case. First, the entire hairpin is deleted in the miRNA mutant strains used for the phenotypic analysis and loss of these miRNAs may have multiple effects on gene expression as they are often predicted to target hundreds of genes (Figure S4 and Table S3). Therefore, it is difficult to foresee what effect on heat stress responses they may have. Second, to mount a response to heat stress, certain TRMs may need to be acutely regulated in adults, for example to control a rapid metabolic change. Therefore, loss of a miRNA may prevent such adaptive possibilities. Thirdly, miRNAs are regulators of genes that may enhance or repress expression and the effect of their expression levels on a specific molecular pathway depends on the function of their targets. Finally, the regulation of multiple TRMs in heat stress may obscure the specific functions of each other or perhaps some TRMs may act in a tissue-specific manner.

Our molecular analysis of HSP and HSF-1 expression showed that their heat stress-induced expression is largely unaffected when individual TRMs are lost. This does not rule out the possibility that the TRMs may play roles in controlling the activity of these proteins via other regulatory factors. Alternatively, the TRMs may act in other pathways or downstream of the HSR pathways we analyzed to enable appropriate heat stress responses.

In conclusion, our high-throughput sequencing strategy identified specific miRNAs that play important roles in heat stress responses in both embryonic development and post-developmental life in *C. elegans.* These miRNAs are predicted to target multiple genes, the expression of which could be modulated to control survival strategies. A combination of miRNAs involved in coordinating these survival responses may ensure that animals are able to combat harsh environmental conditions and generate sufficient progeny to propagate the species.

## Experimental Procedures

### Strains

All *C. elegans* strains generated for this study are detailed in Table S5 and were cultured as previously described^37^ unless otherwise stated.

### Longitudinal Heat Stress Assay at 32°C

Five well-fed adult worms were moved to NGM plates seeded with 300 μl of OP50 bacteria (no more than 72 hrs old) and left at 20°C to produce progeny. From the F1 generation, 10 mid-L4 worms were picked to plates seeded with 50 μl of OP50 bacteria (no more than 72 hrs old). Worms were then incubated for 36–38 hrs at 20°C to reach adulthood. Adult worms were then incubated at 32°C for the indicated timepoints (15, 20 or 25 hrs). At the end of each timepoint, worms were recovered for 24 hrs at 20°C. Following the recovery period, survival was scored by gently prodding with a platinum wire and those animals that failed to respond were scored as dead. >90 worms per timepoint per strain were assayed.

### Longevity at 25°C

Five well-fed L4 worms per strain were moved to a NGM plates and kept in 20°C for about 4 days to produce progeny. 3 × 30–50 adults from the F1 generation were transferred to new plates for a 3 hr egg pulse. 5 × 15 mid-L4 worms from the F2 generation were moved to NGM plates seeded with 300 μl of OP50, 72 h prior to use and incubated for 24 hrs at 20°C to reach adulthood. Young adults were moved to 25°C and were transferred to new plates for the first 4 days of adulthood to prevent overcrowding from progeny. Survival of animals was scored daily. The experiment was performed twice with minimum 41 animals per strain per replicate.

### Egg-laying Experiments

24–72 hrs before the experiment, 6 μl of OP50 bacteria was applied to NGM plates to form a thin layer of bacteria. Single young adult worms were transferred to these plates, were placed at the temperatures indicated (20, 28, 29.5 or 30°C) and incubated for 24 hrs, after which the number of progeny was counted. Progeny from 30 worms per strain was scored.

### Brood Size Experiments

Five well-fed mid-L4 animals per strain were moved from 20°C to 25°C to produce progeny. Five single mid-L4 larvae originating from the F1 generation were moved to individual plates seeded with 50 μl of OP50 24 hrs prior to use. Every 24 hrs each worm was moved to a new plate and the previous plate was left for 24 hrs at 25°C prior to scoring which allows enough time for all the laid eggs to hatch. The experiment was conducted in 3 replicates, 5 worms per replicate. Brood size assessment at 20°C was set up as described above however worms were constantly kept at 20°C.

### Motility Assays

Worms at the L4 stage were transferred to NGM plates seeded with 300 μl of OP50 bacteria and placed at 30°C for 6 hrs. After 6 hrs, single worms were placed on NGM agar fully covered with a lawn of OP50 bacteria (500 μl, no more than five days old) and moved back to 30°C. After 16 hrs, worms were removed from the plates and a grid containing squares was superimposed on the assay plates, and the number of squares entered by the worm tracks was manually counted^38^. 15 worms per strain were assayed.

### RNA Extraction for Small RNA Sequencing

Worms were bead homogenized (6000 rpm, 5 seconds) in a Precellys24 (Precellys, USA) in Trizol LS reagent (Invitrogen, USA). RNA was subsequently extracted according to the Trizol LS protocol (Invitrogen, USA) and co-precipitated with Glycoblue (Ambion, USA) for 30 minutes at −80°C. Subsequently RNA was DNAse treated (RQ1 DNAse, Promega) and was extracted with Acid Phenol Chloroform (Ambion, USA). RNA concentration was measured at a wavelength of 260 nm in a NanoDrop ND-1000 spectrophotometer (NanoDrop Technologies, USA). RNA integrity was determined by capillary gel electrophoresis on a Bioanalyzer (Agilent, USA).

### Small RNA Library Preparation for Sequencing

1 μg of total RNA per sample was used for small RNA library preparation. 5′-monophosphate dependent small RNA cloning and library preparation was then performed with the TruSeq Small RNA Sample Preparation Kit (Illumina, USA). Cluster generation as well as sequencing of the prepared libraries was performed on the Illumina cluster station and GAIIx (Illumina, USA).

### miRNA Fold-change Analysis

Raw sequencing data were preprocessed with a custom PERL script to group reads with different barcodes into separate fasta files. The barcodes used were the following: 35°C 4 h heat stress CGATGT, 20°C control GCCAAT. Next the fasta files were processed with the mapper module from miRDeep2^32^ to clip sequencing adapters, collapse reads and map them to the *C.elegans* genome (WS205) with the following command: mapper.pl config.txt -c -d -i -j -k TGGAATTCTCGGGTGCCAAGG -l 18 -m -p c_elegans.WS205.dna -s reads.fa -t reads_vs_genome.arf -v. Raw read counts as output by the qunatifier module from the miRDeep2 package were normalized to the total number of miRNA reads per barcode then multiplied by the mean value of all miRNA reads of all barcode files. Then a pseudocount of 10 was added and the normalized values were log2 transformed. A regression line with slope 1 was calculated in R for all pairwise sample comparisons and the deviation from each data point to this line (residual) was used to estimate the fold change between two samples.

### RNA Isolation for qRT-PCR

Total RNA including small RNA fraction was isolated in triplicate from staged L4 animals exposed to 20°C, 32°C for 1 hr, 32°C for 2 hrs, 32°C for 4 hrs. Worms were collected with M9 buffer and 1 ml TRIzol® LS Reagent (Life Technologies) was added to app. 0.5 ml worm pellet and stored in −80°C until used. Prior to RNA isolation worms were freeze-cracked by repeated (7x) shifting from liquid nitrogen to 40°C. Total RNA was then isolated following a previously described procedure^39^. Briefly, 200 μl of chloroform was added to 1 ml TRIzol LS-worm lysate and centrifuged for 15 mins, 12.000 g, 4°C to allow phase separation. Aqueous phase containing RNA was transferred to new tube and 1.5 volume of 100% ethanol was added followed by aqueous phase-ethanol mix transfer to miRNeasy column. From this point miRNeasy (Qiagen) protocol was followed to isolate total RNA including miRNAs.

### miRNA Reverse Transcription and qRT-PCR

Universal miRNA cDNA synthesis was performed in triplicate for each of the three biological samples, followed by selective and specific miRNA qRT-PCR according to Ref. 40. Briefly, 100 μg of total RNA was used for one tube poly(A) polymerization and miRNA cDNA synthesis by mixing 1 μl 10 μM universal primer: caggtccagtttttttttttttttvn where V is A, C, G and N is A, C, G, T, 1 μl 10× PAP buffer, 1 μl 1 mM ATP, 1 μl 1 mM dNTPs, 0,5 μl (200 U/ul) MuLV reverse transcriptase (NEB), 0,2 μl (5 U/ul) Poly(A) polymerase and RNAse free water to 10 μl. cDNA was diluted 5× prior to qRT-PCR experiment. miRNA specific primers were designed by the software available at the link below: http://vbn.aau.dk/en/publications/mirprimerdesign3(bcd08649-3883-4016-b493-52dceedd04f8).html

SYBR Green qRT-PCR was performed on The LightCycler® 480 II System (Roche) using 5 μl 2× SYBR Green master mix (Roche), 2 μl RNAse free water, 1 μl of forward, 1 μl of reverse primer (10 μM) and 1 μl cDNA per reaction.

No reverse transcription (noRT) and no template controls (NTC) were included to test for possible DNA contamination. Primer efficiency was assessed by dilution series and dissociation curve was used to assess primer specificity. Two algorithms namely GeNorm and NormFinder were used to assess stability of reference genes after which two most stable reference genes (*mir-86-5p*, *mir-794-5p*) were chosen for further normalization of target genes. Analysis of qRT-PCR data was performed on genEx software (MultiD).

### mRNA Reverse Transcription and qRT-PCR

mRNA cDNA was synthesized in triplicates for each of the three biological samples by a 2 step reaction in which 1 μg of total RNA, 1 μl of a mix of oligodT: Random hexamer 1:3 (100 μM), RNAse free water up to 5 μl were incubated for 5 mins at 70°C and chilled imediately on ice. In second step a master mix consisting of 6.5 μl RNAse free water, 4 μl 5× ImProm II buffer (Promega), 1 μl dNTP mix (10 mM each), 0.5 μl RNasin Ribonuclease inhibitor, 40 u/μl (Promega), 2 μl MgCl_2_ 25 mM, 1 μl ImProm II (Promega) was added to the samples from step 1 and incubated for 5 mins at 25°C, followed by 1 hr at 42°C and inactivation of enzyme at 70°C for 15 mins. cDNA was diluted 5× prior to qRT-PCR experiment. mRNA qRT-PCR was performed and analysed as described in the miRNA qRT-PCR section above, however, for *gpd-1* and *gpd-4* were used as reference genes to normalize mRNA qRT-PCR data.

### Computational Target Prediction

PicTar algorithm^41^ was applied to predict putative mRNAs targeted by miRNAs investigated in the present study. Each prediction is given a score, which determines how reliable the prediction is. The PicTar score is a maximum likelihood score informing how much likely the target site is to be genuine based on a target site model in comparison to a background model. The higher the score the higher confidence that predicted interaction between miRNA and its target mRNA is genuine. In the present study only predictions with PicTar score >1 were included.

## Author Contributions

C.N., A.P., S.M. and K.K. performed the experiments, C.N., A.P. and S.M. performed bioinformatics analysis and C.N., A.P. and R.P. wrote the manuscript.

## Supplementary Material

Supplementary Information Supplementary Information

## Figures and Tables

**Table 1:** Largest miRNA Expression Changes in Heat Stress

miRNA	Fold change	Homology
**Most upregulated at 35°C**		
*mir-83-3p*	4.9	
*mir-239b-5p*	3.7	*mir-12*
*mir-49-3p*	3.3	
*mir-249-3p*	2.2	
*mir-1820-5p*	2.1	
**Most downregulated at 35°C**		
*mir-77-5p*	−7.1	*mir-4647*
*mir-81-5p*	−6.6	
*mir-86-3p*	−5.6	
*mir-51-3p*	−4.9	
*mir-52-3p*	−4.7	
*mir-237-3p*	−3.5	*mir-125*
*mir-58-5p*	−3.2	
*mir-80-5p*	−3.5	*mir-143*
*mir-54-5p*	−3.2	
*mir-84-3p*	−2.5	*let-7*
*mir-74-5p*	−2.2	*mir-185*
*mir-799*	−2.1	
*mir-229-3p*	−2.1	
*mir-71-3p*	−1.9	

Small RNA high-throughput sequencing analysis of control (20°C) and heat stress (35°C) samples revealed 19 miRNAs with >1.9-fold change, based on uniquely mapped, normalized read counts and regression analysis. miRNAs that are members of miRNA families conserved in other phyla are indicated.

## References

[b1] Felix M. A. & Braendle C. The natural history of Caenorhabditis elegans. Curr Biol 20, R965–969, 10.1016/j.cub.2010.09.050 (2010). 21093785

[b2] Braendle C., Milloz J. & Felix M. A. Mechanisms and evolution of environmental responses in Caenorhabditis elegans. Curr Top Dev Biol 80, 171–207, 10.1016/S0070-2153(07)80005-6 (2008). 17950375

[b3] Mohri A. *et al.* Genetic control of temperature preference in the nematode Caenorhabditis elegans. Genetics 169, 1437–1450, 10.1534/genetics.104.036111 (2005). 15654086 PMC1449549

[b4] Golden J. W. & Riddle D. L. The Caenorhabditis elegans dauer larva: developmental effects of pheromone, food, and temperature. Dev Biol 102, 368–378 (1984). 6706004 10.1016/0012-1606(84)90201-x

[b5] Baumeister R., Schaffitzel E. & Hertweck M. Endocrine signaling in Caenorhabditis elegans controls stress response and longevity. The Journal of endocrinology 190, 191–202, 10.1677/joe.1.06856 (2006). 16899554

[b6] Chang A. J. & Bargmann C. I. Hypoxia and the HIF-1 transcriptional pathway reorganize a neuronal circuit for oxygen-dependent behavior in Caenorhabditis elegans. Proc Natl Acad Sci U S A 105, 7321–7326 (2008). 18477695 10.1073/pnas.0802164105 PMC2438248

[b7] Jiang H., Guo R. & Powell-Coffman J. A. The Caenorhabditis elegans hif-1 gene encodes a bHLH-PAS protein that is required for adaptation to hypoxia. Proc Natl Acad Sci U S A 98, 7916–7921 (2001). 11427734 10.1073/pnas.141234698 PMC35443

[b8] Hallem E. A. & Sternberg P. W. Acute carbon dioxide avoidance in Caenorhabditis elegans. Proc Natl Acad Sci U S A 105, 8038–8043, 10.1073/pnas.0707469105 (2008). 18524955 PMC2430355

[b9] Guisbert E., Czyz D. M., Richter K., McMullen P. D. & Morimoto R. I. Identification of a tissue-selective heat shock response regulatory network. PLoS Genet 9, e1003466, 10.1371/journal.pgen.1003466 (2013). 23637632 PMC3630107

[b10] Ogg S. *et al.* The Fork head transcription factor DAF-16 transduces insulin-like metabolic and longevity signals in C. elegans. Nature 389, 994–999 (1997). 9353126 10.1038/40194

[b11] Hsu A. L., Murphy C. T. & Kenyon C. Regulation of aging and age-related disease by DAF-16 and heat-shock factor. Science 300, 1142–1145, 10.1126/science.1083701 (2003). 12750521

[b12] Treinin M. *et al.* HIF-1 is required for heat acclimation in the nematode Caenorhabditis elegans. Physiological genomics 14, 17–24, 10.1152/physiolgenomics.00179.2002 (2003). 12686697

[b13] Scott B. A., Avidan M. S. & Crowder C. M. Regulation of hypoxic death in C. elegans by the insulin/IGF receptor homolog DAF-2. Science 296, 2388-2391 (2002). 12065745 10.1126/science.1072302

[b14] Volovik Y. *et al.* Temporal requirements of heat shock factor-1 for longevity assurance. Aging cell 11, 491–499, 10.1111/j.1474-9726.2012.00811.x (2012). 22360389 PMC4349560

[b15] Bartel D. P. MicroRNAs. Genomics, Biogenesis, Mechanism, and Function. Cell 116, 281–297 (2004). 14744438 10.1016/s0092-8674(04)00045-5

[b16] Bartel D. P. MicroRNAs: target recognition and regulatory functions. Cell 136, 215–233, 10.1016/j.cell.2009.01.002 (2009). 19167326 PMC3794896

[b17] Lee R. C., Feinbaum R. L. & Ambros V. The C. elegans heterochronic gene lin-4 encodes small RNAs with antisense complementarity to lin-14. Cell 75, 843–854 (1993). 8252621 10.1016/0092-8674(93)90529-y

[b18] Pasquinelli A. E. *et al.* Conservation of the sequence and temporal expression of let-7 heterochronic regulatory RNA. Nature 408, 86–89 (2000). 11081512 10.1038/35040556

[b19] Reinhart B. J. *et al.* The 21-nucleotide let-7 RNA regulates developmental timing in Caenorhabditis elegans. Nature 403, 901–906 (2000). 10706289 10.1038/35002607

[b20] Wightman B., Ha I. & Ruvkun G. Posttranscriptional regulation of the heterochronic gene lin-14 by lin-4 mediates temporal pattern formation in C. elegans. Cell 75, 855–862 (1993). 8252622 10.1016/0092-8674(93)90530-4

[b21] Johnston R. J. & Hobert O. A microRNA controlling left/right neuronal asymmetry in Caenorhabditis elegans. Nature 426, 845–849 (2003). 14685240 10.1038/nature02255

[b22] Pedersen M. E. *et al.* An epidermal microRNA regulates neuronal migration through control of the cellular glycosylation state. Science 341, 1404–1408, 10.1126/science.1242528 (2013). 24052309

[b23] Boulias K. & Horvitz H. R. The C. elegans MicroRNA mir-71 Acts in Neurons to Promote Germline-Mediated Longevity through Regulation of DAF-16/FOXO. Cell Metab 15, 439–450, 10.1016/j.cmet.2012.02.014 (2012). 22482727 PMC3344382

[b24] Shaw W. R., Armisen J., Lehrbach N. J. & Miska E. A. The conserved miR-51 microRNA family is redundantly required for embryonic development and pharynx attachment in Caenorhabditis elegans. Genetics 185, 897–905, 10.1534/genetics.110.117515 (2010). 20421599 PMC2900971

[b25] Shen Y., Wollam J., Magner D., Karalay O. & Antebi A. A steroid receptor-microRNA switch regulates lifespan in response to signals from the gonad. Science 338, 1472–1476, 10.1126/science.1228967 (2012). 23239738 PMC3909774

[b26] de Lencastre A. *et al.* MicroRNAs both promote and antagonize longevity in C. elegans. Curr Biol 20, 2159–2168, 10.1016/j.cub.2010.11.015 (2010). 21129974 PMC3023310

[b27] Vora M. *et al.* Deletion of microRNA-80 activates dietary restriction to extend C. elegans healthspan and lifespan. PLoS Genet 9, e1003737, 10.1371/journal.pgen.1003737 (2013). 24009527 PMC3757059

[b28] Brenner J. L., Jasiewicz K. L., Fahley A. F., Kemp B. J. & Abbott A. L. Loss of individual microRNAs causes mutant phenotypes in sensitized genetic backgrounds in C. elegans. Curr Biol 20, 1321–1325, 10.1016/j.cub.2010.05.062 (2010). 20579881 PMC2946380

[b29] Boehm M. & Slack F. A developmental timing microRNA and its target regulate lifespan in C. elegans. Science 310, 1954–1957, 10.1126/science.1115596 (2005). 16373574

[b30] Miska E. A. *et al.* Most Caenorhabditis elegans microRNAs Are Individually Not Essential for Development or Viability. PLoS Genet 3, e215 (2007). 18085825 10.1371/journal.pgen.0030215 PMC2134938

[b31] Friedlander M. R. *et al.* Discovering microRNAs from deep sequencing data using miRDeep. Nature biotechnology 26, 407–415, 10.1038/nbt1394 (2008). 18392026

[b32] Friedlander M. R., Mackowiak S. D., Li N., Chen W. & Rajewsky N. miRDeep2 accurately identifies known and hundreds of novel microRNA genes in seven animal clades. Nucleic Acids Res 40, 37–52, 10.1093/nar/gkr688 (2012). 21911355 PMC3245920

[b33] Van Raamsdonk J. M. *et al.* Decreased energy metabolism extends lifespan in Caenorhabditis elegans without reducing oxidative damage. Genetics 185, 559–571, 10.1534/genetics.110.115378 (2010). 20382831 PMC2881137

[b34] Johnson T. E. *et al.* Gerontogenes mediate health and longevity in nematodes through increasing resistance to environmental toxins and stressors. Exp Gerontol 35, 687–694 (2000). 11053658 10.1016/s0531-5565(00)00138-8

[b35] McMullen P. D. *et al.* Macro-level modeling of the response of C. elegans reproduction to chronic heat stress. PLoS Comput Biol 8, e1002338, 10.1371/journal.pcbi.1002338 (2012). 22291584 PMC3266876

[b36] McColl G. *et al.* Insulin-like signaling determines survival during stress via posttranscriptional mechanisms in C. elegans. Cell Metab 12, 260–272, 10.1016/j.cmet.2010.08.004 (2010). 20816092 PMC2945254

[b37] Brenner S. The genetics of Caenorhabditis elegans. Genetics 77, 71–94 (1974). 4366476 10.1093/genetics/77.1.71 PMC1213120

[b38] Flavell S. W. *et al.* Serotonin and the neuropeptide PDF initiate and extend opposing behavioral states in C. elegans. Cell 154, 1023–1035, 10.1016/j.cell.2013.08.001 (2013). 23972393 PMC3942133

[b39] Cirera S. Highly efficient method for isolation of total RNA from adipose tissue. BMC research notes 6, 472, 10.1186/1756-0500-6-472 (2013). 24245791 PMC4225616

[b40] Balcells I., Cirera S. & Busk P. K. Specific and sensitive quantitative RT-PCR of miRNAs with DNA primers. BMC biotechnology 11, 70, 10.1186/1472-6750-11-70 (2011). 21702990 PMC3135530

[b41] Lall S. *et al.* A genome-wide map of conserved microRNA targets in C. elegans. Curr Biol 16, 460–471 (2006). 16458514 10.1016/j.cub.2006.01.050

