# Author Correction: Specific microRNAs Regulate Heat Stress Responses in *Caenorhabditis elegans*

**DOI:** 10.1038/s41598-019-43688-4

**Published:** 2019-05-20

**Authors:** Camilla Nehammer, Agnieszka Podolska, Sebastian D. Mackowiak, Konstantinos Kagias, Roger Pocock

**Affiliations:** 1 Biotech Research and Innovation Centre, University of Copenhagen, Ole Maaløes Vej 5, Copenhagen, Denmark; 2 Max-Delbrück-Center for Molecular Medicine, Robert-Rössle-Straße 10, 13125 Berlin, Germany; 3 Department of Anatomy and Developmental Biology, Faculty of Biomedical and Psychological Sciences, Monash University, Clayton Victoria, Australia

Correction to: *Scientific Reports* 10.1038/srep08866, published online 9 March 2015.

This Article contains errors.

The raw sequencing data was not originally included with the article and is no longer available. The pre-processed data with a description of processing is now publicly deposited. As such, the corrected Data Availability section should read:

**Data Availability**

The pre-processed sequencing data and the description of the processing are included in files deposited with Open Science Framework and is available at https://osf.io/7t8j9.

