# Supplementary material for: Specific microRNAs Regulate Heat Stress Responses in Caenorhabditis elegans
Source: Sci Rep. 2015 Mar 9;5:8866. doi: 10.1038/srep08866 (PMC4352874; doi:10.1038/srep08866)
Supplement: Supplementary Information [file srep08866-s1.pdf]

Specific microRNAs Regulate Heat Stress Responses in *Caenorhabditis elegans*

Camilla Nehammer[1*], Agnieszka Podolska[1*], Sebastian D. Mackowiak[2], Konstantinos Kagias[1] and Roger Pocock[1, 3]

[1]Biotech Research and Innovation Centre, University of Copenhagen, Ole Maaløes Vej 5, Copenhagen, Denmark

[2]Max-Delbrück-Center for Molecular Medicine, Robert-Rössle-Straße 10, 13125, Berlin

[3]Department of Anatomy and Developmental Biology, Faculty of Biomedical and Psychological Sciences, Monash University, Clayton, Victoria, Australia.

* equal contributors

Correspondence should be addressed to R.P.

Department of Anatomy and Developmental Biology, Faculty of Biomedical and Psychological Sciences, Monash University, Clayton, Victoria, Australia.

Tel: +61 3 990 50654

email: roger.pocock@monash.edu

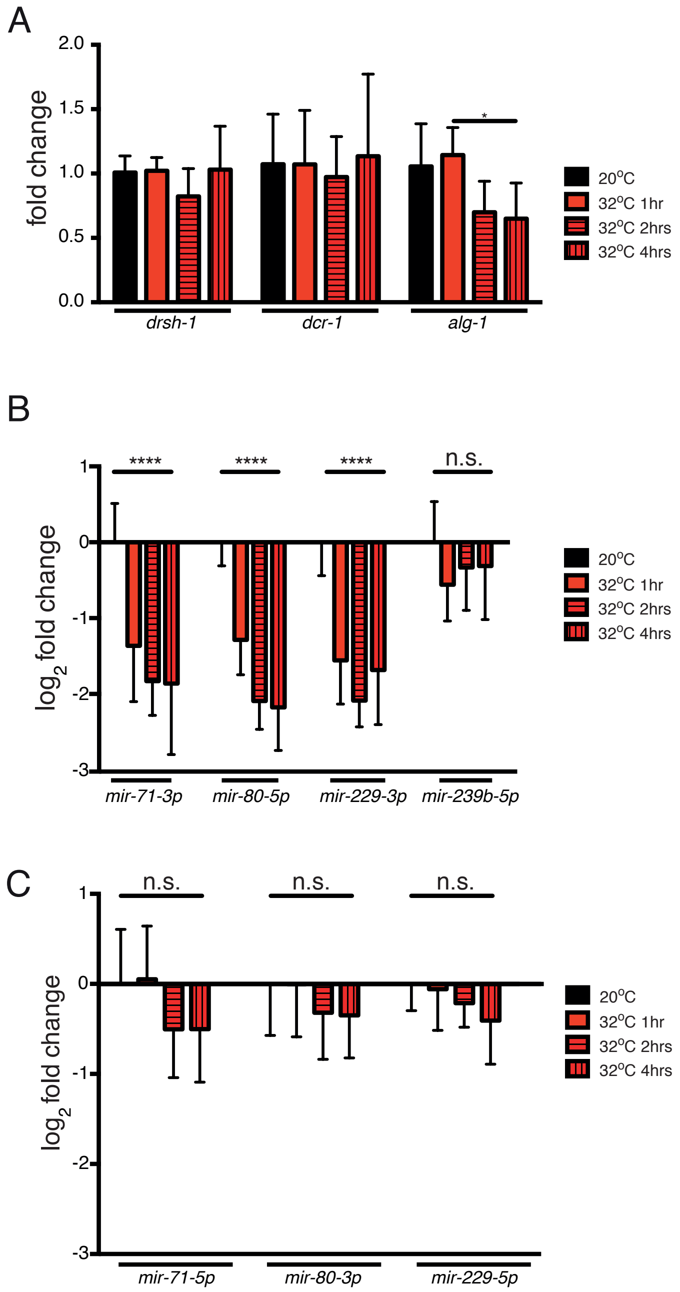

Figure S1. qRT-PCR Analysis

(A) miRNA biogenesis machinery factors *drsh-1*, *dcr-1* and *alg-1* are not markedly thermoregulated. Data was normalized to two reference mRNAs namely *gpd-1* and *gpd-4* and are presented as mean fold changes ± SEM. Statistical significance was assessed by two-way ANOVA analysis followed by Tukey's multiple comparisons test. *p<0.05.

(B-C) *mir-71-3p*, *mir-80-5p*, *mir-229-3p* are rapidly thermoregulated at 32°C, whereas *mir-239b-5p* is not (B). miRNAs opposite to those shown to be regulated in RNA sequencing

are not thermoregulated (C). *mir-239b3p* is not presented on the figure as it was below the level of detection (C). Data was normalized to two reference miRNAs namely *mir-86-5p* and *mir-794-5p* and are presented as log2 means ± SEM. Statistical significance was assessed by two-way ANOVA analysis followed by Tukey's multiple comparisons test. ****$p<0.0001$, n.s - not significant.

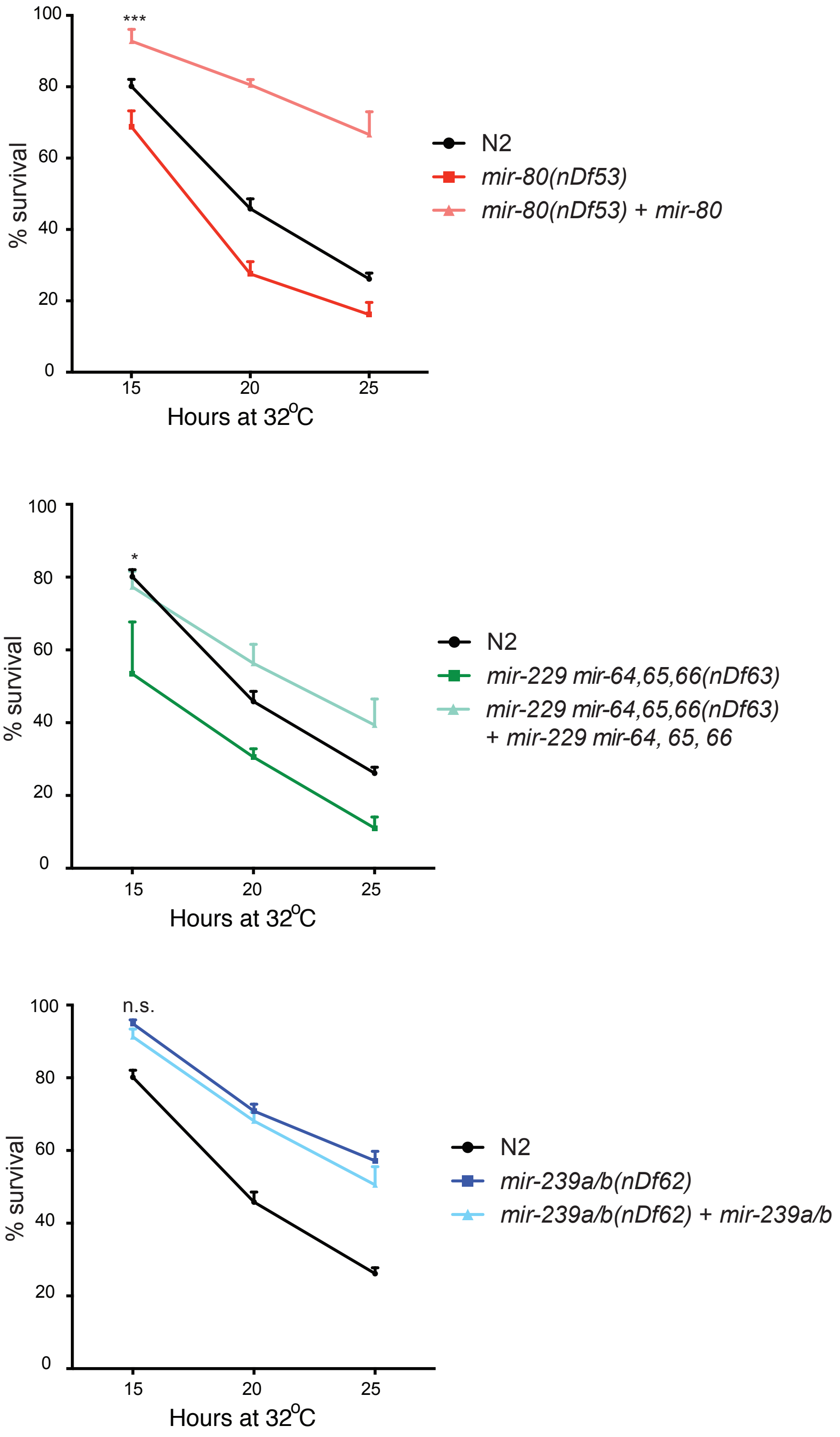

Figure S2. Rescue of miRNA Heat Stress Phenotypes

(a-c) Heat stress phenotypes of *mir-80(nDf53)* and *mir-229, 64-66(nDf63)* are rescued by transgenic expression of the respective miRNAs under the control of their own regulatory sequences. We were unable to rescue the *mir-239a/b(nDf62)* heat resistance phenotype.

One representative rescue line is shown for each, three lines were scored - data not shown. Data are presented as means ± SEM and statistical significance was assessed by 2way ANOVA followed by Tukey's multiple comparison test. $n>110$,
*$p<0.05$, ***$p<0.001$, n.s. - not significant.

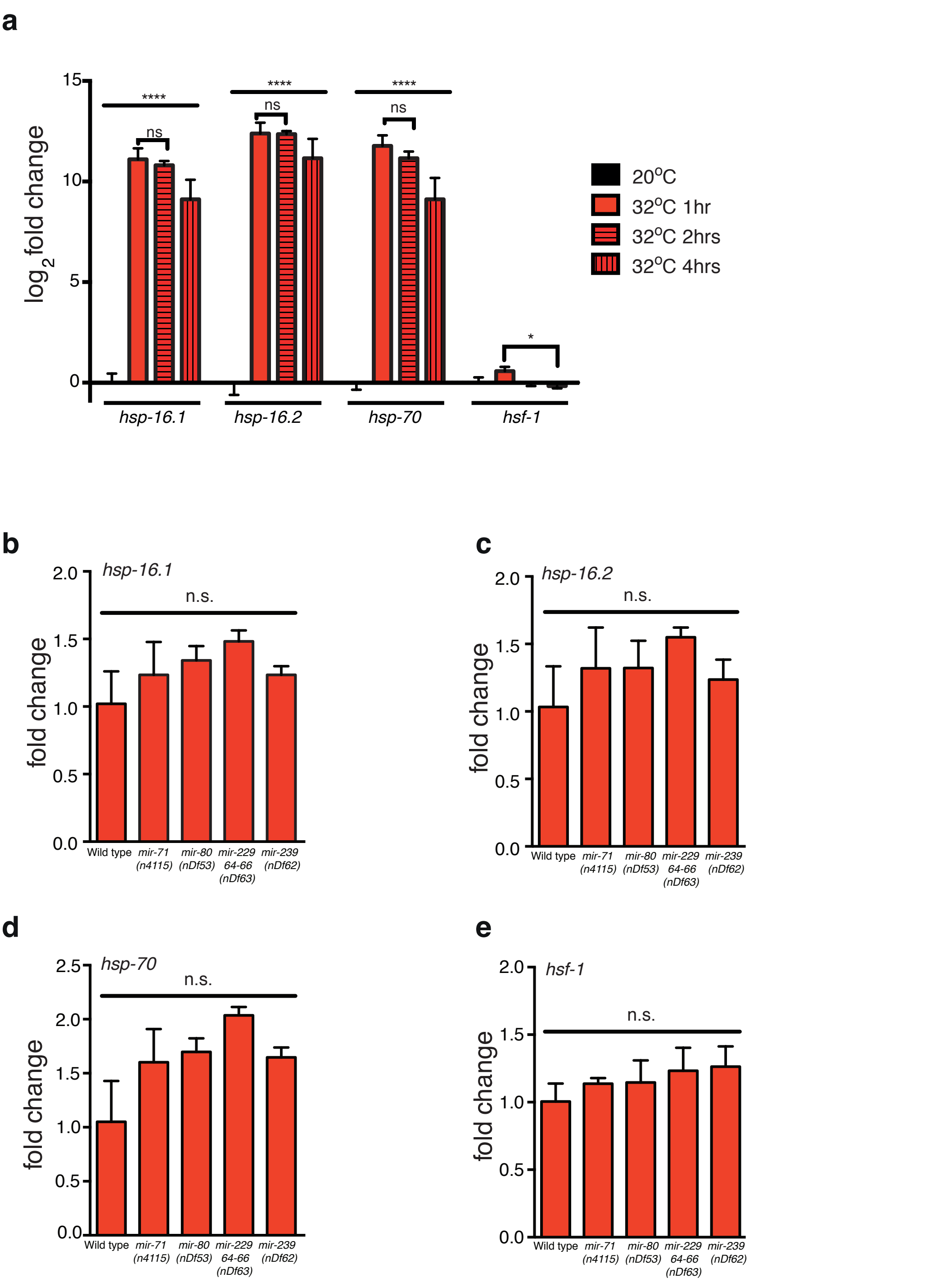

Figure S3. Molecular Analysis of Thermoregulated miRNA Function

(a) qRT-PCR analysis showing relative expression levels of *hsp-16.1*, *hsp-16.2*, *hsp-70* and *hsf-1* in wild type animals at 20°C and after a 32°C heat stress for 1, 2 and 4 hrs. (b-e) qRT-PCR analysis showing relative expression levels of *hsp-16.1*, *hsp-16.2*, *hsp-70* and *hsf-1* in TRM mutant backgrounds.

Data are normalized to two reference genes, *gpd-1* and *gpd-4* in A; *pmp-3* and *cdc-42* in (a-e); and are presented as log2 means ± SEM (a) and means ± SEM (b-e). Statistical significance was assessed by two-way ANOVA analysis followed by Tukey's multiple comparisons test. Three independent biological replicates were used for RNA isolation.

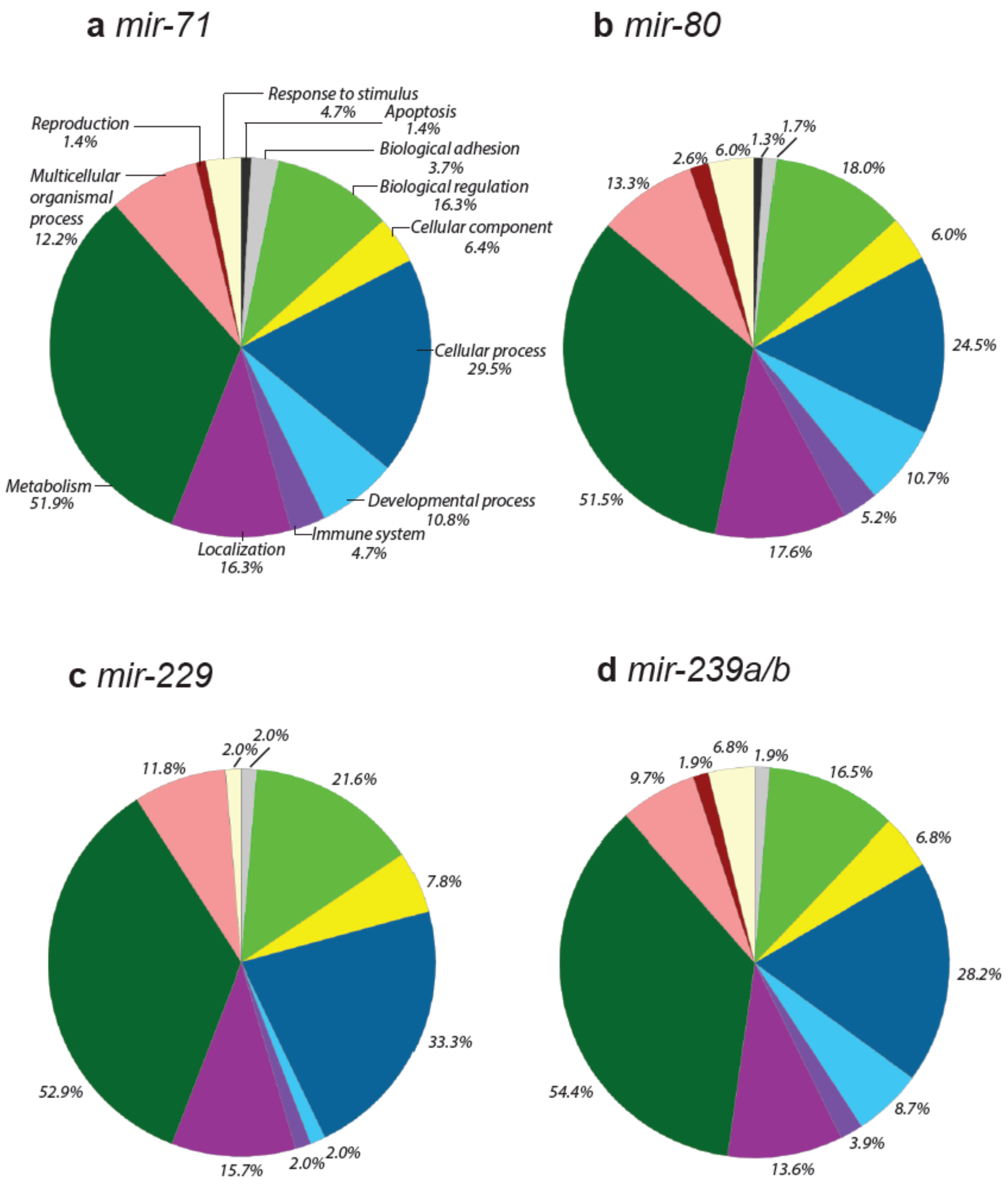

Figure S4. The majority of predicted targets for *mir-71*, *mir-80*, *mir-229* and *mir-239* are genes involved in metabolic processes

(a-d) Pie charts of the broad biological functions of predicted miRNA targets for *mir-71*, *mir-80*, *mir-229* and *mir-239*. Biological functions were categorized using the PANTHER classification system and results are depicted as percent of process hits against total number of hits, a number of the predicted targets fall into multiple categories.

miRNAs regulated by heat stress
Read counts >150 and fold change >1.9

miRNA	Fold change	Normalized read counts in 35°C	Normalized read counts in 20°C	Sequence
mir-83-3p	4.901	1387.692	339.952	UAGCACCAUAUAAAUUCAGUAA
mir-239b-5p	3.772	13328.593	4300.273	UUUGUACUACACAAAAGUACUG
mir-49-3p	3.398	480.841	167.802	AAGCACCACGAGAAGCUGCAGA
mir-249-3p	2.270	454.128	241.705	UCACAGGACUUUUGAGCGUUGCC
mir-1820-5p	2.101	538.486	311.261	UUUUGAUUGUUUUUCGAUGAUGUUCG
mir-77-5p	-7.089	600.349	5312.306	GAUGGUUGUGCUCUGAGGAAAU
mir-81-5p	-6.644	185.588	1593.691	CGGUUUUCACCGUGAUCUGAGA
mir-86-3p	-5.618	281.193	2006.678	CUGGGCUCAGAUUCGCUUAGGC
mir-52-3p	-4.789	239.014	1460.666	CACGUUACAAUGAAAGGGUAGC
mir-237-3p	-3.599	206.677	952.041	CUGUCGAGUUUUGUCAAGGACC
mir-80-5p	-3.574	4501.915	19706.831	AGCUUUCGACAUGAUUCUGAAC
mir-54-5p	-3.269	1259.748	5085.381	AGGAUAUGAGACGACGAGAACA
mir-58-5p	-3.251	627.062	2537.039	UGCCCUACUCUUCGCAUCUCA
mir-84-3p	-2.561	392.265	1258.955	CACAAUGUUUCAACUAACUCGGC
mir-74-5p	-2.248	160.280	462.544	CGGGCUUCCAUCUCUUUCCCAGC
mir-799	-2.128	178.558	485.150	UGAACCCUGAUAAAGCUAGUGG
mir-229-3p	-2.105	3420.724	8830.079	AGAAAGGUAUCGGGUGUCAUAG
mir-71-3p	-1.959	2056.933	4954.095	UAUCACUAUUCUGUUUUUCGC

miRNAs from the opposite strands to those regulated by heat stress

miRNA	Fold change	Normalized read counts in 35°C	Normalized read counts in 20°C	Sequence
mir-80-3p	1.632	104000.991	77189.291	UGAGAUCAUUAGUUGAAAGCCGA
mir-239b-3p	n.a.	2.811	0.000	GCACUUUUGUGGUGUGCAAAAA
mir-54-3p	1.396	6730.377	5887.009	UACCCGUAAUCUUCAUAAUCCGAG
mir-1820-3p	1.354	5.623	4.347	AACCAUUGUAAACAAUCAAAGA
mir-84-5p	1.334	15451.608	14112.824	UGAGGUAGUAUGUAAUAUUGUAGA
mir-81-3p	1.309	501027.178	461700.298	UGAGAUCAUCGUGAAAGCUAGU
mir-74-3p	1.270	376.799	365.166	UGAGAUCGUUCAGUACGGCAAU
mir-52-5p	1.238	20231.905	19899.848	CACCCGUACAUAUGUUUCCGUGCU
mir-49-5p	-1.573	172.934	345.169	CGCAGUUUGUUGUGAUGUGCUCC
mir-83-5p	-1.470	14.059	33.908	ACUGAAUUUAUGUGUGUACUUGA
mir-77-3p	-1.218	8160.248	12134.838	UUCAUCAGGCCAUAGCUGUCCA
mir-86-5p	-1.218	6436.529	6457.365	UAAGUGAAUGCUUUGCCACAGUC
mir-237-5p	-1.192	98214.020	99827.020	UCCCUGAGAAUUCUCGAACAGCU
mir-249-5p	n.a.	0.000	0.860	AGCAACGCACAAACGUCUUCUGUG
mir-58-3p	-1.125	349938.852	375488.779	UGCCCUACUCUUCGCAUCUCA
mir-71-5p	-1.123	10685.370	11601.868	UGAAAGACAUGGGUAGUGAGACG
mir-229-5p	-1.096	38831.479	43064.460	AAUGACACUGGUUAUCUUUUCCAUCG
mir-799	n.a.	n.a.	n.a.	n.a.

Table S1. miRNA Sequencing

Table listing miRNAs with >150 normalized read counts, regulated >2-fold according to regression analysis and corresponding miRNAs originating from opposite strands, lacking regulation in heat stress. Data analysis was performed by miRDeep2 [31,32].

Strain	Number of animals (n)	Median lifespan (days)	Mean lifespan (days)	Maximal lifespan (days)	p value against N2	% of mean lifespan change against N2	comments
**2 trials combined**							
N2	97	10	10.8	19		NA	
mir-71	91	8	7.6	13	< 0.0001	29.63	p value based on survival curve
mir-80	101	9	10.3	17.5	n.s.	4.63	
mir-229	114	7	8.3	16	< 0.0001	23.15	p value based on survival curve
mir-239	98	10	10.1	16	n.s.	6.48	
**trial 1**							
N2	42	8	9.6	19		NA	
mir-71	41	7	7.1	12		26.04	
mir-80	52	9	9.4	16		2.08	
mir-229	57	7	7.5	17		21.88	
mir-239	42	8	9.3	14		3.12	
**trial 2**							
N2	55	11	11.7	19		NA	
mir-71	50	8	8	14		31.62	
mir-80	49	12	11.3	19		3.42	
mir-229	57	8	8.8	15		24.79	
mir-239	56	11	11.2	18		4.27	

Table S2. Lifespan Analysis at 25°C

C. elegans miRNA mutant strains backcrossed to N2 three times were assayed at 25°C. Life span analysis was repeated twice (n>90-118) with similar effects. Mean life span values and statistical analyses of life span assays are shown. Data are presented as means ± SEM and statistical significance was assessed by 2way ANOVA followed by Tukey's multiple comparison test. n>110, *p<0.05, **p<0.01,****p<0.0001, n.s. - not significant.

Target predictions for miRNAs regulated in RNAseq

**mir-71-3p**	**PicTar score**	
vha-15	7,0828	required for systemic RNAi, probably during endocytotic RNAi uptake
F55C12.1	5,547	-
mec-2	4,1877	mechanical stimuli
pmk-3	4,0565	MAPK pathways are involved in stress response and innate immunity
che-12	3,8608	Involveed in converting environmental stimuli into neuronal activity
otub-1	3,5318	unknown
R07G3.8	3,5134	unknown
Y9C2UA.1	3,4858	unknown
tram-1	3,2744	unknown
saps-1	3,2176	required for proper spindle positioning in embryoes
T10B9.9	3,1354	unknown
Y106G6G.4	3,1043	Homology to cell wall mannoprotein
Y57G11C.38	3,0901	unknown
mlc-3	3,0719	activity is required for both embryonic and postembryonic development
nol-5	3,0548	Homology to protein involved in pre-rRNA processing
vha-12	3,0001	suppresses necrotic neurodegeneration
dlat-1	2,9843	-
ZK652.8	2,9652	unknown
nhr-10	2,717	predicted as a nuclear hormone receptor
nos-2	2,6601	nos-2 affects incorporation of primordial germ cells into the somatic gonad
flp-32	2,4339	unknown
RIT3P.239	2,3482	RIT3P.239
nmy-1	2,2331	required for embryonic elongation and establishment of normal body morphology
F42G8.10	1,9847	unknown
T14B1.1	1,8882	unknown
F43G9.3	1,6074	homology to mitochondrial carrier protein
C10G11.6	1,5017	unknown
wdfy-2	1,4856	essential for wild-type levels of endocytosis
dnj-1	1,3428	homology of protein which is a co-chaperone for Hsp40
F49H12.5	1,1724	

**mir-80-5p**	**PicTar score**	
rpl-10	4,4451	required for embryonic viability
hpo-26	2,8216	unknown
lin-14	2,5154	required for specifying the division timings of cells during postembryonic development
rpl-17	2,3675	activity is required for embryonic and germline development
mdt-28	2,2071	unknown
pab-2	1,529	pab-2 encodes a polyadenylate-binding protein 1 homolog
rsp-7	1,0777	required for constitutive splicing and influence alternative splicing regulation.

**mir-229-3p**	**PicTar score**	
cutl-16	7,2138	unknown
peb-1	5,9701	required for proper morphogenesis of the pharynx, vulva
unc-130	3,2383	member of the forkhead domain family of transcription factors
ran-1	3,2085	transport of proteins into nucleus
eif-3.C	3,0867	encodes a putative c subunit of translation initiation factor 3 that is required for fertility
sqv-5	2,8202	encodes a chondroitin synthase that both initiates and elongates chondroitin chains
sas-5	2,6646	required for centrosome duplication in C. elegans
C02D5.2	2,1529	unknown
pat-9	1,8232	nuclear zinc finger protein critical for the assembly of muscle attachments
F17C11.10	1,7794	unknown
lron-1	1,7783	homology to leucine-rich repeat-containing G-protein coupled receptor 5
T23B12.11	1,646	unknown
byn-1	1,4897	homolog of mammalian BYSTIN-LIKE
F13D12.9	1,3538	unknown
C34F6.7	1,1971	unknown
ncs-2	1,0461	Neuronal Calcium Sensor family

**mir-239b-5p**	**PicTar score**	
F53C11.4	7,5134	unknown
rpa-4	7,3299	unknown
nlp-24	6,5062	Neuropeptide-Like Protein
sta-2	5,6183	unknown
K11D9.3	5,4969	unknown
mnk-1	5,3609	mnk-1 encodes the C. elegans ortholog of the Mnk MAP kinase-interacting kinase
nprl-3	4,7594	unknown
C15C8.7	4,5803	unknown
Y53F4B.14	4,3808	unknown
R151.4	4,1425	homology to Fibroblast growth factor receptor
hecd-1	4,0372	HECtD1 ubiquitin ligase homolog
hsp-12.1	4,0325	member of the small heat shock family of proteins
fat-1	4,0217	encodes an omega-3 fatty acyl desaturase
pfn-1	3,9521	required for embryonic cytokinesis
mdt-15	3,8211	MDT-15 participates in basal and fasting-induced transcription of numerous other metabolic genes

Target predictions for miRNAs regulated in RNAseq

**mir-71-3p**	**PicTar score**	
vha-15	7,0828	required for systemic RNAi, probably during endocytotic RNAi uptake
F55C12.1	5,547	-
mec-2	4,1877	mechanical stimuli
pmk-3	4,0565	MAPK pathways are involved in stress response and innate immunity
che-12	3,8608	Involveed in converting environmental stimuli into neuronal activity
otub-1	3,5318	unknown
R07G3.8	3,5134	unknown
Y9C2UA.1	3,4858	unknown
tram-1	3,2744	unknown
saps-1	3,2176	required for proper spindle positioning in embryoes
T10B9.9	3,1354	unknown
Y106G6G.4	3,1043	Homology to cell wall mannoprotein
Y57G11C.38	3,0901	unknown
mlc-3	3,0719	activity is required for both embryonic and postembryonic development
nol-5	3,0548	Homology to protein involved in pre-rRNA processing
vha-12	3,0001	suppresses necrotic neurodegeneration
dlat-1	2,9843	-
ZK652.8	2,9652	unknown
nhr-10	2,717	predicted as a nuclear hormone receptor
nos-2	2,6601	nos-2 affects incorporation of primordial germ cells into the somatic gonad
flp-32	2,4339	unknown
RIT3P.239	2,3482	RIT3P.239
nmy-1	2,2331	required for embryonic elongation and establishment of normal body morphology
F42G8.10	1,9847	unknown
T14B1.1	1,8882	unknown
F43G9.3	1,6074	homology to mitochondrial carrier protein
C10G11.6	1,5017	unknown
wdfy-2	1,4856	essential for wild-type levels of endocytosis
dnj-1	1,3428	homology of protein which is a co-chaperone for Hsp40
F49H12.5	1,1724	

**mir-80-5p**	**PicTar score**	
rpl-10	4,4451	required for embryonic viability
hpo-26	2,8216	unknown
lin-14	2,5154	required for specifying the division timings of cells during postembryonic development
rpl-17	2,3675	activity is required for embryonic and germline development
mdt-28	2,2071	unknown
pab-2	1,529	pab-2 encodes a polyadenylate-binding protein 1 homolog
rsp-7	1,0777	required for constitutive splicing and influence alternative splicing regulation.

**mir-229-3p**	**PicTar score**	
cutl-16	7,2138	unknown
peb-1	5,9701	required for proper morphogenesis of the pharynx, vulva
unc-130	3,2383	member of the forkhead domain family of transcription factors
ran-1	3,2085	transport of proteins into nucleus
eif-3.C	3,0867	encodes a putative c subunit of translation initiation factor 3 that is required for fertility
sqv-5	2,8202	encodes a chondroitin synthase that both initiates and elongates chondroitin chains
sas-5	2,6646	required for centrosome duplication in C. elegans
C02D5.2	2,1529	unknown
pat-9	1,8232	nuclear zinc finger protein critical for the assembly of muscle attachments
F17C11.10	1,7794	unknown
lron-1	1,7783	homology to leucine-rich repeat-containing G-protein coupled receptor 5
T23B12.11	1,646	unknown
byn-1	1,4897	homolog of mammalian BYSTIN-LIKE
F13D12.9	1,3538	unknown
C34F6.7	1,1971	unknown
ncs-2	1,0461	Neuronal Calcium Sensor family

**mir-239b-5p**	**PicTar score**	
F53C11.4	7,5134	unknown
rpa-4	7,3299	unknown
nlp-24	6,5062	Neuropeptide-Like Protein
sta-2	5,6183	unknown
K11D9.3	5,4969	unknown
mnk-1	5,3609	mnk-1 encodes the C. elegans ortholog of the Mnk MAP kinase-interacting kinase
nprl-3	4,7594	unknown
C15C8.7	4,5803	unknown
Y53F4B.14	4,3808	unknown
R151.4	4,1425	homology to Fibroblast growth factor receptor
hecd-1	4,0372	HECtD1 ubiquitin ligase homolog
hsp-12.1	4,0325	member of the small heat shock family of proteins
fat-1	4,0217	encodes an omega-3 fatty acyl desaturase
pfn-1	3,9521	required for embryonic cytokinesis
mdt-15	3,8211	MDT-15 participates in basal and fasting-induced transcription of numerous other metabolic genes

Target predictions for miRNAs regulated in RNAseq

**mir-71-3p**	**PicTar score**	
vha-15	7,0828	required for systemic RNAi, probably during endocytotic RNAi uptake
F55C12.1	5,547	-
mec-2	4,1877	mechanical stimuli
pmk-3	4,0565	MAPK pathways are involved in stress response and innate immunity
che-12	3,8608	Involveed in converting environmental stimuli into neuronal activity
otub-1	3,5318	unknown
R07G3.8	3,5134	unknown
Y9C2UA.1	3,4858	unknown
tram-1	3,2744	unknown
saps-1	3,2176	required for proper spindle positioning in embryoes
T10B9.9	3,1354	unknown
Y106G6G.4	3,1043	Homology to cell wall mannoprotein
Y57G11C.38	3,0901	unknown
mlc-3	3,0719	activity is required for both embryonic and postembryonic development
nol-5	3,0548	Homology to protein involved in pre-rRNA processing
vha-12	3,0001	suppresses necrotic neurodegeneration
dlat-1	2,9843	-
ZK652.8	2,9652	unknown
nhr-10	2,717	predicted as a nuclear hormone receptor
nos-2	2,6601	nos-2 affects incorporation of primordial germ cells into the somatic gonad
flp-32	2,4339	unknown
RIT3P.239	2,3482	RIT3P.239
nmy-1	2,2331	required for embryonic elongation and establishment of normal body morphology
F42G8.10	1,9847	unknown
T14B1.1	1,8882	unknown
F43G9.3	1,6074	homology to mitochondrial carrier protein
C10G11.6	1,5017	unknown
wdfy-2	1,4856	essential for wild-type levels of endocytosis
dnj-1	1,3428	homology of protein which is a co-chaperone for Hsp40
F49H12.5	1,1724	

**mir-80-5p**	**PicTar score**	
rpl-10	4,4451	required for embryonic viability
hpo-26	2,8216	unknown
lin-14	2,5154	required for specifying the division timings of cells during postembryonic development
rpl-17	2,3675	activity is required for embryonic and germline development
mdt-28	2,2071	unknown
pab-2	1,529	pab-2 encodes a polyadenylate-binding protein 1 homolog
rsp-7	1,0777	required for constitutive splicing and influence alternative splicing regulation.

**mir-229-3p**	**PicTar score**	
cutl-16	7,2138	unknown
peb-1	5,9701	required for proper morphogenesis of the pharynx, vulva
unc-130	3,2383	member of the forkhead domain family of transcription factors
ran-1	3,2085	transport of proteins into nucleus
eif-3.C	3,0867	encodes a putative c subunit of translation initiation factor 3 that is required for fertility
sqv-5	2,8202	encodes a chondroitin synthase that both initiates and elongates chondroitin chains
sas-5	2,6646	required for centrosome duplication in C. elegans
C02D5.2	2,1529	unknown
pat-9	1,8232	nuclear zinc finger protein critical for the assembly of muscle attachments
F17C11.10	1,7794	unknown
lron-1	1,7783	homology to leucine-rich repeat-containing G-protein coupled receptor 5
T23B12.11	1,646	unknown
byn-1	1,4897	homolog of mammalian BYSTIN-LIKE
F13D12.9	1,3538	unknown
C34F6.7	1,1971	unknown
ncs-2	1,0461	Neuronal Calcium Sensor family

**mir-239b-5p**	**PicTar score**	
F53C11.4	7,5134	unknown
rpa-4	7,3299	unknown
nlp-24	6,5062	Neuropeptide-Like Protein
sta-2	5,6183	unknown
K11D9.3	5,4969	unknown
mnk-1	5,3609	mnk-1 encodes the C. elegans ortholog of the Mnk MAP kinase-interacting kinase
nprl-3	4,7594	unknown
C15C8.7	4,5803	unknown
Y53F4B.14	4,3808	unknown
R151.4	4,1425	homology to Fibroblast growth factor receptor
hecd-1	4,0372	HECtD1 ubiquitin ligase homolog
hsp-12.1	4,0325	member of the small heat shock family of proteins
fat-1	4,0217	encodes an omega-3 fatty acyl desaturase
pfn-1	3,9521	required for embryonic cytokinesis
mdt-15	3,8211	MDT-15 participates in basal and fasting-induced transcription of numerous other metabolic genes

Target predictions for miRNAs regulated in RNAseq

**mir-71-3p**	**PicTar score**	
vha-15	7,0828	required for systemic RNAi, probably during endocytotic RNAi uptake
F55C12.1	5,547	-
mec-2	4,1877	mechanical stimuli
pmk-3	4,0565	MAPK pathways are involved in stress response and innate immunity
che-12	3,8608	Involveed in converting environmental stimuli into neuronal activity
otub-1	3,5318	unknown
R07G3.8	3,5134	unknown
Y9C2UA.1	3,4858	unknown
tram-1	3,2744	unknown
saps-1	3,2176	required for proper spindle positioning in embryoes
T10B9.9	3,1354	unknown
Y106G6G.4	3,1043	Homology to cell wall mannoprotein
Y57G11C.38	3,0901	unknown
mlc-3	3,0719	activity is required for both embryonic and postembryonic development
nol-5	3,0548	Homology to protein involved in pre-rRNA processing
vha-12	3,0001	suppresses necrotic neurodegeneration
dlat-1	2,9843	-
ZK652.8	2,9652	unknown
nhr-10	2,717	predicted as a nuclear hormone receptor
nos-2	2,6601	nos-2 affects incorporation of primordial germ cells into the somatic gonad
flp-32	2,4339	unknown
RIT3P.239	2,3482	RIT3P.239
nmy-1	2,2331	required for embryonic elongation and establishment of normal body morphology
F42G8.10	1,9847	unknown
T14B1.1	1,8882	unknown
F43G9.3	1,6074	homology to mitochondrial carrier protein
C10G11.6	1,5017	unknown
wdfy-2	1,4856	essential for wild-type levels of endocytosis
dnj-1	1,3428	homology of protein which is a co-chaperone for Hsp40
F49H12.5	1,1724	

**mir-80-5p**	**PicTar score**	
rpl-10	4,4451	required for embryonic viability
hpo-26	2,8216	unknown
lin-14	2,5154	required for specifying the division timings of cells during postembryonic development
rpl-17	2,3675	activity is required for embryonic and germline development
mdt-28	2,2071	unknown
pab-2	1,529	pab-2 encodes a polyadenylate-binding protein 1 homolog
rsp-7	1,0777	required for constitutive splicing and influence alternative splicing regulation.

**mir-229-3p**	**PicTar score**	
cutl-16	7,2138	unknown
peb-1	5,9701	required for proper morphogenesis of the pharynx, vulva
unc-130	3,2383	member of the forkhead domain family of transcription factors
ran-1	3,2085	transport of proteins into nucleus
eif-3.C	3,0867	encodes a putative c subunit of translation initiation factor 3 that is required for fertility
sqv-5	2,8202	encodes a chondroitin synthase that both initiates and elongates chondroitin chains
sas-5	2,6646	required for centrosome duplication in C. elegans
C02D5.2	2,1529	unknown
pat-9	1,8232	nuclear zinc finger protein critical for the assembly of muscle attachments
F17C11.10	1,7794	unknown
lron-1	1,7783	homology to leucine-rich repeat-containing G-protein coupled receptor 5
T23B12.11	1,646	unknown
byn-1	1,4897	homolog of mammalian BYSTIN-LIKE
F13D12.9	1,3538	unknown
C34F6.7	1,1971	unknown
ncs-2	1,0461	Neuronal Calcium Sensor family

**mir-239b-5p**	**PicTar score**	
F53C11.4	7,5134	unknown
rpa-4	7,3299	unknown
nlp-24	6,5062	Neuropeptide-Like Protein
sta-2	5,6183	unknown
K11D9.3	5,4969	unknown
mnk-1	5,3609	mnk-1 encodes the C. elegans ortholog of the Mnk MAP kinase-interacting kinase
nprl-3	4,7594	unknown
C15C8.7	4,5803	unknown
Y53F4B.14	4,3808	unknown
R151.4	4,1425	homology to Fibroblast growth factor receptor
hecd-1	4,0372	HECtD1 ubiquitin ligase homolog
hsp-12.1	4,0325	member of the small heat shock family of proteins
fat-1	4,0217	encodes an omega-3 fatty acyl desaturase
pfn-1	3,9521	required for embryonic cytokinesis
mdt-15	3,8211	MDT-15 participates in basal and fasting-induced transcription of numerous other metabolic genes

Target predictions for miRNAs regulated in RNAseq

**mir-71-3p**	**PicTar score**	
vha-15	7,0828	required for systemic RNAi, probably during endocytotic RNAi uptake
F55C12.1	5,547	-
mec-2	4,1877	mechanical stimuli
pmk-3	4,0565	MAPK pathways are involved in stress response and innate immunity
che-12	3,8608	Involveed in converting environmental stimuli into neuronal activity
otub-1	3,5318	unknown
R07G3.8	3,5134	unknown
Y9C2UA.1	3,4858	unknown
tram-1	3,2744	unknown
saps-1	3,2176	required for proper spindle positioning in embryoes
T10B9.9	3,1354	unknown
Y106G6G.4	3,1043	Homology to cell wall mannoprotein
Y57G11C.38	3,0901	unknown
mlc-3	3,0719	activity is required for both embryonic and postembryonic development
nol-5	3,0548	Homology to protein involved in pre-rRNA processing
vha-12	3,0001	suppresses necrotic neurodegeneration
dlat-1	2,9843	-
ZK652.8	2,9652	unknown
nhr-10	2,717	predicted as a nuclear hormone receptor
nos-2	2,6601	nos-2 affects incorporation of primordial germ cells into the somatic gonad
flp-32	2,4339	unknown
RIT3P.239	2,3482	RIT3P.239
nmy-1	2,2331	required for embryonic elongation and establishment of normal body morphology
F42G8.10	1,9847	unknown
T14B1.1	1,8882	unknown
F43G9.3	1,6074	homology to mitochondrial carrier protein
C10G11.6	1,5017	unknown
wdfy-2	1,4856	essential for wild-type levels of endocytosis
dnj-1	1,3428	homology of protein which is a co-chaperone for Hsp40
F49H12.5	1,1724	

**mir-80-5p**	**PicTar score**	
rpl-10	4,4451	required for embryonic viability
hpo-26	2,8216	unknown
lin-14	2,5154	required for specifying the division timings of cells during postembryonic development
rpl-17	2,3675	activity is required for embryonic and germline development
mdt-28	2,2071	unknown
pab-2	1,529	pab-2 encodes a polyadenylate-binding protein 1 homolog
rsp-7	1,0777	required for constitutive splicing and influence alternative splicing regulation.

**mir-229-3p**	**PicTar score**	
cutl-16	7,2138	unknown
peb-1	5,9701	required for proper morphogenesis of the pharynx, vulva
unc-130	3,2383	member of the forkhead domain family of transcription factors
ran-1	3,2085	transport of proteins into nucleus
eif-3.C	3,0867	encodes a putative c subunit of translation initiation factor 3 that is required for fertility
sqv-5	2,8202	encodes a chondroitin synthase that both initiates and elongates chondroitin chains
sas-5	2,6646	required for centrosome duplication in C. elegans
C02D5.2	2,1529	unknown
pat-9	1,8232	nuclear zinc finger protein critical for the assembly of muscle attachments
F17C11.10	1,7794	unknown
lron-1	1,7783	homology to leucine-rich repeat-containing G-protein coupled receptor 5
T23B12.11	1,646	unknown
byn-1	1,4897	homolog of mammalian BYSTIN-LIKE
F13D12.9	1,3538	unknown
C34F6.7	1,1971	unknown
ncs-2	1,0461	Neuronal Calcium Sensor family

**mir-239b-5p**	**PicTar score**	
F53C11.4	7,5134	unknown
rpa-4	7,3299	unknown
nlp-24	6,5062	Neuropeptide-Like Protein
sta-2	5,6183	unknown
K11D9.3	5,4969	unknown
mnk-1	5,3609	mnk-1 encodes the C. elegans ortholog of the Mnk MAP kinase-interacting kinase
nprl-3	4,7594	unknown
C15C8.7	4,5803	unknown
Y53F4B.14	4,3808	unknown
R151.4	4,1425	homology to Fibroblast growth factor receptor
hecd-1	4,0372	HECtD1 ubiquitin ligase homolog
hsp-12.1	4,0325	member of the small heat shock family of proteins
fat-1	4,0217	encodes an omega-3 fatty acyl desaturase
pfn-1	3,9521	required for embryonic cytokinesis
mdt-15	3,8211	MDT-15 participates in basal and fasting-induced transcription of numerous other metabolic genes

Table S3. miRNA Target Predictions

Target predictions for: 1) Thermoregulated miRNAs and 2) miRNAs on the opposite strand to that regulated by heat stress.

qRT-PCR oligonucleotides

Transcript	Forward primer	Reverse primer
mir-71-3p	cgcagtatcactattctgt	ccagttttttttttttttttgcga
mir-71-5p	agtgaaagacatgggtagtg	tccagttttttttttttttttcgtc
mir-80-3p	cgcagtgagatcattagttg	gttttttttttttttttcggctttc
mir-80-5p	cagagctttcgacatgattc	tccagttttttttttttttttgttcag
mir-229-3p	gagaaaggtatcgggtgtc	tccagttttttttttttttttctatgac
mir-229-5p	gaatgacactggttatcttttcc	tccagttttttttttttttttcgatg
mir-239b-3p	gcaggcacttttgtgg	tccagttttttttttttttttgcac
mir-239b-5p	cgcagtttgtactacaca	gtccagttttttttttttttttcagt
mir-86-5p	agtaagtgaatgctttgcca	gtccagttttttttttttttttgact
mir-794-5p	cagtgaggtaatcatcgttgt	gtccagttttttttttttttttagtga
drsh-1	ccacttccggattgtatgac	gtcgaaacactcatcaggc
dcr-1	tagtgatttagtctcgctctcg	cacccaatttcaaagtctttgc
alg-1	tcctggaggaactattcaacac	gctgataatctcgcgattaactc
hsp-16.1	ggctcagatggaacgtcaa	tggcaaacttttgatcattgtta
hsp-16.2	tgcagaatctctccatctgagt	tggtttaaactgtgagacgttga
hsp-70	cggtatttatcaaaatggaaaggtt	tacgagcggcttgatctttt
hsf-1	tcagacagttgaatatgtacgg	cctgatctgattctgttcgag
gpd-1	atcacgttgtttctaacgcatc	atgagtccttcgatgataccg
gpd-4	tcacgttatttctaacgcatcg	atgagtccttcgatgataccg

Genotyping oligonucleotides

Mutant allele	Forward primer	Reverse primer	Amplicon size (bp)	Reverse primer - wild type band only	Amplicon size (bp)
MT12993 *mir-71(n4115)*	ggaaggaacagttacatagtgc	cactggatgacgttaggttaagc	wild type-520 mutant-460	NA	NA
MT13949 *mir-80(nDf53)*	gcatccaatcgagcgcag	NA	NA	cacacggacggactgcc	592
MT16494 *mir-229, mir-64-66(nDf63)*	cgtcaaatcggcacattgcc	NA	NA	ggggatccacacagagaatc	543
MT15312 *mir-239a/b(nDf62)*	ggagtagcaccagtggg	NA	NA	ggtacagtggcaagaagacaca	876

Rescue PCR oligonucleotides

Mutant allele	Forward primer	Reverse primer	Amplicon size (bp)
RJP999 *mir-229, mir-64-66(nDf63)*	ggagtctgagccaatattctagc	ggagtctgagccaatattctagc	3396
RJP1014 *mir-239a/b(nDf62)*	catgcgttcgtcttcttcc	cctaatgcaaccgtcttcaa	2208
RJP936 *mir-80(nDf53)*	gattctccaaagaacccattgc	ggtcatcggaggacatcac	2377

Table S4. List of Oligonucleotides Used in This Study

Strain name	miRNA_gene	Chromosome	allele	# outcrossed
MT12993	*mir-71*	I	*n4115*	0x
RJP907	*mir-71*	I	*n4115*	3x
MT13949	*mir-80*	III	*nDf53*	0x
RJP936	*mir-80*	III	*nDf53*	3x
MT16494	*mir-229, mir-64-66*	III	*nDf63*	2x
RJP999	*mir-229, mir-64-66*	III	*nDf63*	5x
MT15312	*mir-239a/b*	X	*nDf62*	0x
RJP1014	*mir-239a/b*	X	*nDf62*	3x
RJP1742	rpEx741 [mir-80;myo-2::mCherry]; mir-80(nDf53)III	III	*nDf53*	n/a
RJP1661	rpEx710 (mir-229;myo-2::mCherry);mir-229, 64, 65, 66(nDf63)III	III	*nDf63*	n/a
RJP1741	rpEx740 [mir-239a mir-239b;myo-2::mCherry]; mir-239a mir-239b (nDf62) X	X	*nDf62*	n/a

Table S5. List of Strains Used in This Study
